# Survival of *Desulfotomaculum* spores from estuarine sediments after serial autoclaving and high-temperature exposure

**DOI:** 10.1038/ismej.2014.190

**Published:** 2014-10-17

**Authors:** Louise A O'Sullivan, Erwan G Roussel, Andrew J Weightman, Gordon Webster, Casey RJ Hubert, Emma Bell, Ian Head, Henrik Sass, R John Parkes

**Affiliations:** 1 School of Earth and Ocean Sciences, Cardiff University, Cardiff CF10 3AT, Wales, UK; 2 Cardiff School of Biosciences, Cardiff University, Cardiff CF10 3AT, Wales, UK; 3 School of Civil Engineering and Geosciences, Newcastle University, Newcastle upon Tyne NE1 7RU, UK

## Abstract

Bacterial spores are widespread in marine sediments, including those of thermophilic, sulphate-reducing bacteria, which have a high minimum growth temperature making it unlikely that they grow *in situ*. These *Desulfotomaculum* spp. are thought to be from hot environments and are distributed by ocean currents. Their cells and spores upper temperature limit for survival is unknown, as is whether they can survive repeated high-temperature exposure that might occur in hydrothermal systems. This was investigated by incubating estuarine sediments significantly above (40–80 °C) maximum *in situ* temperatures (∼23 °C), and with and without prior triple autoclaving. Sulphate reduction occurred at 40–60 °C and at 60 °C was unaffected by autoclaving. *Desulfotomaculum* sp. C1A60 was isolated and was most closely related to the thermophilic *D. kuznetsovii*^T^ (∼96% 16S rRNA gene sequence identity). Cultures of *Desulfotomaculum* sp. C1A60, *D. kuznetsovii*^T^and *D. geothermicum* B2T survived triple autoclaving while other related *Desulfotomaculum* spp. did not, although they did survive pasteurisation. *Desulfotomaculum* sp. C1A60 and *D. kuznetsovii* cultures also survived more extreme autoclaving (C1A60, 130 °C for 15 min; *D. kuznetsovii*, 135 °C for 15 min, maximum of 154 °C reached) and high-temperature conditions in an oil bath (C1A60, 130° for 30 min, *D. kuznetsovii* 140 °C for 15 min). *Desulfotomaculum* sp. C1A60 with either spores or predominantly vegetative cells demonstrated that surviving triple autoclaving was due to spores. Spores also had very high culturability compared with vegetative cells (∼30 × higher). Combined extreme temperature survival and high culturability of some thermophilic *Desulfotomaculum* spp. make them very effective colonisers of hot environments, which is consistent with their presence in subsurface geothermal waters and petroleum reservoirs.

## Introduction

Bacterial endospores can withstand multiple environmental stress conditions, such as ionizing radiation, heat, pressure, desiccation, pH extremes and toxic chemicals and are thus remarkable survival systems (Reineke *et al.*, 2013), able to persist over geological timescales (Cano and Borucki, 1995). Spores are widespread in marine waters and sediments (Muller *et al.*, 2014) and may become relatively more abundant with sediment depth (Fichtel *et al.*, 2008), with numbers possibly equivalent to those of vegetative cells (Lomstein *et al.*, 2012). Spore-forming bacteria have also been isolated from sub-seafloor sediments (for example, Inagaki *et al.*, 2003; D'Hondt *et al.*, 2004; Suss *et al.*, 2004) including Gram-positive, spore-forming, thermophilic, sulphate-reducing bacteria (Aullo *et al.*, 2013) who's spores have been shown to have a half-life of ∼300 years in some sediments (de Rezende *et al.*, 2013). It has been suggested that thermophilic spores from warm subsurface petroleum reservoirs and ocean crust ecosystems may be constantly delivered to cold marine sediments via seeps and seawater, and might be able to germinate as the sediment warms during burial (Hubert *et al.*, 2009, 2010). The spores in seawater may also be drawn down through sediments, inoculating them, and then carried along the basalt basement, as part of the ocean aquifer system that feeds hydrothermal vents, and migrate upwards in fractures and faults to inoculate the sediments above (Parkes *et al.*, 2014). In hydrothermal systems spores may initially experience temperatures and pressures that exceed their thermophilic growth limit before reaching conditions suitable for growth. Hence, it would be interesting to know the upper temperature and pressure limits that sedimentary spores can survive and how this might vary between different spore-forming bacteria, such as different *Desulfotomaculum* species. In addition, considering the dynamic nature of hydrothermal systems it would be informative to know whether spores could survive repeated heating cycles with the potential development of superdormant spores (Ghosh *et al.*, 2009). Any enhanced heat stability and ability to withstand multiple high-temperature–pressure cycles would also have implications for sterility in general, and autoclaved controls used in experiments to distinguish between biogenic and thermogenic processes in deep sediments.

The survival temperatures for spore-forming, sulphate-reducing bacteria was addressed during an investigation of the impact of minerals on deep sedimentary biogeochemical processes (Parkes *et al.*, 2011) as previously triple-autoclaved, abiological control sediment slurries (Portishead, Severn Estuary, tidal mud flats, UK) showed active sulphate reduction at 60 °C. The bacteria from these slurries were enriched and isolated and their ability to survive triple autoclaving as both vegetative cells and spores was determined. The maximum autoclave conditions, maximum temperature and range for growth and survival were also determined, and compared with other *Desulfotomaculum* strains, including *D. kuznetsovii*^T^ a close relative of the sediment slurry isolate, but from underground (2800–3250 m) thermal mineral waters (Nazina *et al.*, 1989). To demonstrate that this phenomenon was not restricted to sediments from a single location, we also determined the survival of sulphate-reducing bacteria in sediment slurries from the Tyne and Tamar estuaries, UK, following triple autoclaving.

## Materials and methods

### The effect of autoclaving on sulphate reduction in sediment slurries

**Figure 1:**
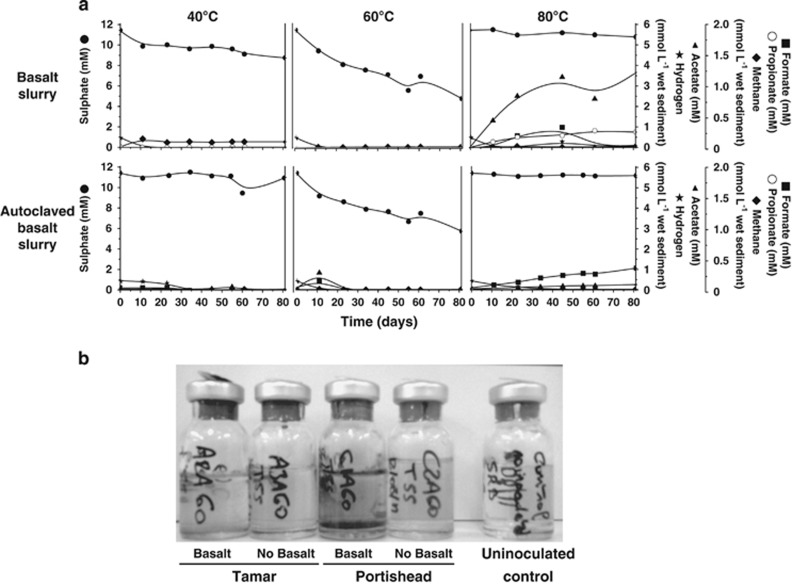
(**a**) Effect of autoclaving Portishead sediment slurries and incubation at 40, 60 and 80 °C, and (**b**) subsequent enrichment of autoclaved slurries at 60 °C along with Tamar Estuary sediment slurries similarly treated. Left to right: A2A60 Tamar slurry with basalt—negative; A3A60 Tamar slurry no basalt—negative; C1A60 Portishead slurry with basalt—positive; C2A60 Portishead slurry without basalt—negative; and uninoculated control—negative.

Severn Estuary tidal mud flat sediments (Portishead, 7–51 cm depth) were slurried with degassed artificial seawater (pH 7.6, 25% slurry, 2 l total volume, (Wellsbury *et al.*, 1994) and 1% (v/v) Guaymas Basin hydrothermal sediment added as an additional prokaryotic inoculum. Ground basalt (100 g l^−1^, Parkes *et al.*, 2011) was added, as a mineral addition, and dispensed anaerobically in 10-ml aliquots in crimp vials in an anaerobic cabinet flushed with N_2_. Replicate vials were autoclaved three times at 121 °C for 20 min, before being incubated at 40, 60 and 80 °C for up to 100 days. At discrete time intervals (Figure 1) vials were sacrificed for headspace and pore water analysis. At the end of the experiment slurries with active sulphate reduction were subcultured into marine sulphate-reducing bacterial medium with 2 mM fatty acid mix (see below) at 60 °C. Similar experiments were conducted with Tamar Estuary, UK, sediments. In addition, sediment from the Tyne Estuary, UK (collected at low tide from the black anoxic zone at 20–40 cm depth), were slurried (2:1 v/w, pH 7.1, 20 mM sulphate) with degassed brackish mineral medium (Widdel and Bak, 1992). Acetate, propionate, butyrate, lactate and glucose were added to a final concentration of 3 mM, and tryptic soy broth to 3 g l^−1^ (source of nutrients, vitamins, minerals and additional substrates). This slurry was then dispensed into 60-ml crimp vials under a constant flow of N_2_ and sealed with butyl rubber stoppers. Triplicates were autoclaved three times successively as described above before incubation at 50 and 80 °C for 14 days.

### *Desulfotomaculum* spp. used in this study

Repeated agar shake tubes (Parkes *et al.*, 2009) were used to isolate pure cultures from a positive subculture of an autoclaved Portishead sediment slurry (C1A60). Two individual colonies were picked and subcultured, but as these showed no differences in appearance and in initial physiological and autoclaving tests, they were considered identical and further experiments were conducted using one of them, *Desulfotomaculum* sp. C1A60.

A number of additional *Desulfotomaculum* spp. were investigated for comparison, representing both surface and subsurface species (Table 1). *D. kuznetsovii* DSM6115^T^ (Nazina *et al.*, 1989) and *D. thermosapovorans* DSM6562^T^ (Fardeau *et al.*, 1995) were obtained from the Deutsche Sammlung von Mikroorganismen und Zellkulturen (DSMZ, Braunschweig, Germany). *D. acetoxidans* DSM771^T^ (Widdel and Pfennig, 1977), *D. geothermicum* B2T (Sass and Cypionka, 2004) and *Desulfotomaculum* sp. NC402 (Sass *et al.*, 2003) were from our own culture collection. Growth conditions, temperature range for growth and origin of the strains are given in Table 1.

### Growth media and substrates

For enrichment, isolation and pure-culture experiments two anoxic mineral media were used: (1) an artificial seawater medium containing the following components in g l^−1^: NaCl (24.3), MgCl_2_·6H_2_O (10), CaCl_2_·2H_2_O (1.5), KCl (0.66), Na_2_SO_4_ (1.4), KBr (0.1), H_3_BO_3_ (0.0025), SrCl_2_·6H_2_O (0.04), NH_4_Cl (0.021), KH_2_PO_4_ (0.0054) and NaF (0.003), and (2) a freshwater medium containing (g l^−1^): NaCl (0.25), MgCl_2_·6H_2_O (0.25), CaCl_2_·2H_2_O (0.1), KCl (0.1), Na_2_SO_4_ (1.4), NH_4_Cl (0.1) and KH_2_PO_4_ (0.1). Both media were supplemented with 1 ml l^−1^ of trace-element solution SL 10 and 0.2 ml l^−1^ of selenite tungstate solution (Widdel and Bak, 1992), and 0.5 ml l^−1^ of a resazurin solution (0.5 mg ml^−1^). After autoclaving, the media were cooled under an atmosphere of N_2_/CO_2_ (80:20, vol/vol), prior to addition of 10 ml l^−1^ vitamin solution (Wolin *et al.*, 1963) and 30 ml l^−1^ sodium bicarbonate solution (1 M) from sterile stock solutions. When a visual indication of sulphate reduction by black FeS formation was required, 0.5 ml l^−1^ FeSO_4_ (1 M in dilute H_2_SO_4_, autoclaved under an N_2_ atmosphere) was added. Media was reduced by the addition of a few sterile crystals of sodium dithionite, and the pH adjusted to between 7.2 and 7.4 by addition of sterile HCl or Na_2_CO_3_, if necessary.

Substrates were added from sterile stocks before inoculation or as a headspace of H_2_/CO_2_ (80/20 vol/vol). The fatty acid mix (FA mix) stock solution contained sodium formate, acetate, propionate, butyrate, valerate and caproate, each at 1 M.

### Physiology of *Desulfotomaculum* sp. C1A60

Growth and utilization of substrates was tested in dithionite-reduced medium in completely filled screw-cap tubes, or in vials or tubes sealed with butyl rubber stoppers with a H_2_/CO_2_ or N_2_/CO_2_ headspace. Positive tests were subcultured to ensure that growth was not due to substrate carry over. Growth of *Desulfotomaculum* sp. C1A60 at different temperatures was determined in a thermal gradient system (Barnes *et al.*, 1998). Three growth substrates were tested: (A) no organic substrates plus H_2_/CO_2_ atmosphere; (B) 2 mM fatty acid mix plus H_2_/CO_2_ atmosphere; and (C) 2 mM fatty acid mix plus N_2_/CO_2_ atmosphere. The medium was inoculated with 5% (vol/vol) of a *Desulfotomaculum* sp. C1A60 culture that had been grown with 20 mM sodium lactate and contained no microscopically visible spores or signs of sporulation. In an anaerobic cabinet, 9.5 ml of the appropriate pre-inoculated medium was aliquoted into sterile glass vials and sealed with butyl rubber septum stoppers. After removal from the cabinet, the vials were crimped and gassed with either H_2_/CO_2_ or N_2_/CO_2_ to a positive pressure. Vials were incubated for 28 days in the thermal gradient system between 16 and 100 °C and then growth determined by microscopy. Concentrations of sulphate, lactate and fatty acids were determined by ion chromatography (Parkes *et al.*, 2011).

### Triple-autoclave experiments with *Desulfotomaculum* spp

Cultures were assessed microscopically to confirm that they contained spores, and cultures that contained the highest number of spores were selected. However, spore numbers were not standardised for different inoculating cultures and were quite variable. Two different containers were used to detect any potential effect of gas permeability during autoclaving: 15 ml non-gas-tight autoclavable conical plastic tubes (BD Falcon, Oxford, UK) and gas impermeable anaerobic glass tubes with butyl rubber septum stoppers (Bellco, SciQuip, Newtown, UK). In an anaerobic cabinet, 5 ml of culture was aliquoted into tubes, which were then autoclaved in an upright position (121 °C for 15 min) once, twice or three times. Repeated autoclave runs were started immediately when the autoclave cooled down to ∼30 °C after the previous run. Autoclaved tubes were transferred to an anaerobic cabinet, and 750 μl used to inoculate a 50-ml bottle of medium. Replicate bottles were generally screened microscopically after 2 weeks. Negative tubes were further incubated and then re-scored. *D. acetoxidans* was routinely incubated longer as its growth was slow. Uninoculated media blanks were used as negative controls for each strain. Where pasteurization was carried out it was for 15 min at 75 °C. Correct autoclave function was confirmed with Merck Sterikon plus Bioindicator autoclaving controls (Merck, Darmstadt, Germany), VWR temperature indicator papers (VWR, Lutterworth, UK, ±1 °C) and 3 M Comply Thermalog Steam Chemical Integrator (3 M, Bracknell, UK).

### Elevated autoclave and temperature survival experiments

Survival of *Desulfotomaculum* sp. C1A60 and *D. kuznetsovii*^T^ at temperatures above 121 °C was tested in an autoclave and in an oil bath, for consistent and accurate temperatures (Model ONE 45, Memmert, Schwabach, Germany, temperature stability ±0.3 °C). All cultures were set up in an anaerobic cabinet. For autoclave experiments, 3 ml of inoculum was aliquoted into either a 15-ml Falcon tube or an anaerobic Bellco vial with a butyl rubber septum. After autoclaving, 750 μl was used to inoculate a 50-ml bottle of medium. For experiments using the oil bath, 3 ml of the inoculating culture was aliquoted into 30-ml glass serum bottles that were sealed with butyl rubber septum stoppers and crimped. The sealed serum bottles were placed between two fixed metal plates during heating. After heating, either 750 μl was removed from the serum bottle with a syringe and used to inoculate a 50-ml bottle of medium, or 15 ml medium was injected directly into the serum bottle.

### *Desulfotomaculum* sp. C1A60 viability of spores and vegetative cells after autoclaving

Vegetative cell-only cultures were grown for about 2 weeks at 58 °C with 20 mM lactate as substrate. Microscopic examination showed that the culture contained dividing cells but no spores or evidence of sporulation. The spore-only suspension was prepared by gastrografin density-gradient centrifugation as previously described (Fichtel *et al.*, 2007), and the purified spores were stored in 0.9% NaCl at 4 °C under air, as extended oxygen exposure had previously been found to kill *Desulfotomaculum* sp. C1A60 vegetative cells. Therefore, any remaining vegetative cells were considered to be non-viable. Microscopic counts of these original untreated suspensions were conducted under coverslips on agar-coated slides. Spore and vegetative cell suspensions were aliquoted (5 × 2 ml each) into sterile crimp tubes in the anaerobic cabinet and stored at 4 °C overnight. The following treatments were then performed: no treatment, pasteurization (20 min at 85 °C in water bath), single, double and triple autoclaving (15 min at 121 °C each time). Tubes were again stored at 4 °C overnight before inoculating two parallel most probable number (MPN) series for each treated culture. MPN series were set up in an anaerobic cabinet in 96-deep-well plates (Kopke *et al.*, 2005) using the standard marine dithionite-reduced media containing 2 mM fatty acid mix and 0.5 mM FeCl_2_. MPN plates were placed in plastic bags containing a catalyst to ensure an anoxic atmosphere and closed using a plastic clip (Anaerocult A mini, VWR). Because of the elevated incubation temperature, 60 °C, the bags were additionally heat-sealed. MPN series were incubated for 17 days. Wells were scored for growth by both visual (black FeS formation) and microscopic examination.

### *Desulfotomaculum* sp. C1A60 16S rRNA and dsrA gene sequences

Genomic DNA was extracted from a *Desulfotomaculum* sp. C1A60 cell pellet, obtained by centrifugation of a 50-ml culture, using the FastDNA Spin Kit for soil (MP Biomedicals, Santa Ana, CA, USA) and eluted in 100 μl molecular biology grade water (Severn Biotech Ltd, Kiddeminster, UK). The 16S rRNA and dissimilatory sulphite reductase (*dsrAB*) genes were amplified by PCR with primers 27F/1492R (Lane, 1991) and DSR1F/DSR4R (Wagner *et al.*, 1998), respectively, using GoTaq Flexi DNA polymerase (Promega Corporation, Southampton, UK) and conditions previously described (Wagner *et al.*, 1998; Webster *et al.*, 2006). The *dsrAB* PCR product was then purified (QIAquick PCR Purification Kit, Qiagen, Manchester, UK), and sequenced directly with primers DSR1F, DSR4R and DSR3R (Wagner *et al.*, 1998) by Eurofins Genomics (Wolverhampton, UK). However, the 16S rRNA gene could not be sequenced directly because of multiple gene copies. Therefore, triplicate PCR reactions were pooled, purified and concentrated (Amicon Ultra Centrifugal filters; Millipore Ltd, Darmstadt, Germany) before being cloned (pGEM-T Easy Vector System I; Promega Corporation) and transformed into *Escherichia coli* JM109 competent cells according to the manufacturer's instructions. Colony PCR with transformed cell biomass was then used directly with M13 primers (O'Sullivan *et al.*, 2008) and individual clone 16S rRNA gene products were sequenced (Eurofins Genomics).

All sequence chromatograms were viewed and edited using Chromas Lite software version 2.1.1 (http://www.technelysium.com.au), and 16S rRNA and *dsrAB* gene consensus sequences were produced from overlapping sequences using BioEdit Sequence Alignment Editor version 7.2.0 (Hall, 1999). Closest sequence matches were identified using the nucleotide blast (blastn) suite at the National Center for Biotechnology Information (NCBI; http://www.ncbi.nlm.nih.gov).

### Tyne Estuary sediment slurry 16S rRNA gene sequence and comparative phylogenetic analysis

DNA was extracted from Tyne Estuary sediment slurries using the MoBio PowerSoil DNA Isolation Kit (Cambio Ltd., Cambridge, UK). Extracted DNA was amplified using primers 27F/1492R as above and PCR products were cloned using TOPO TA Cloning Kit (Life Technologies Ltd., Paisley, UK) according to the manufacturer's instructions. Clone inserts were amplified with vector primers pUCF (5′-GTTTTCCCAGTCACGAC-3′) and M13R and sequenced (Genevision, Newcastle Upon Tyne, UK). The 16S rRNA gene sequence from the Tyne Estuary sediment slurries, *Desulfotomaculum* sp. C1A60 clones and other representative *Desulfotomaculum* spp., including those used in this study, were aligned using ClustalX2 (Larkin *et al.*, 2007), trimmed in BioEdit and phylogenetic trees were constructed using Minimum Evolution and LogDet distance method in MEGA version 6 (Tamura *et al.*, 2013). Congruent trees were also obtained using other methods including neighbour-joining with Jukes–Cantor algorithm, maximum likelihood with the Tamura–Nei model and Maximum Parsimony.

### Nucleotide sequence accession numbers

All new nucleotide sequences determined in this study have been deposited in the GenBank database under accession numbers KM065442 to KM065444 for *Desulfotomaculum* sp. C1A60 16S rRNA genes, KM065445 for *Desulfotomaculum* sp. C1A60 *dsrA* gene and KM208880 for the Tyne Estuary sediment slurry bacterium 16S rRNA gene.

## Results and discussion

### Extremely heat-resistant *Desulfotomaculum* spores in temperate estuarine sediments

In Portishead non-autoclaved sediment slurries there was no sulphate reduction at 80 °C, despite the formation of volatile fatty acids. However, clear sulphate removal occurred at both 40 and 60 °C, with the rate at 60 °C being much faster than at 40 °C (Figure 1). This was surprising as the average sea-surface temperature at this location is ∼12 °C, with a maximum of ∼23 °C, hence, the thermophilic prokaryotes responsible for sulphate reduction at 60 °C would probably not be active at *in situ* temperatures. Even more surprising sulphate removal still occurred at 60 °C, even after the slurry was triple autoclaved before incubation (Figure 1). In comparison, no significant sulphate reduction occurred in triple-autoclaved slurries incubated at either 40 or 80 °C. These results were reproducible (data not shown). However, identical experiments with Tamar Estuary sediment slurries also showed sulphate reduction at both 40 and 60 °C, but no activity after autoclaving (unpublished results).

**Figure 2:**
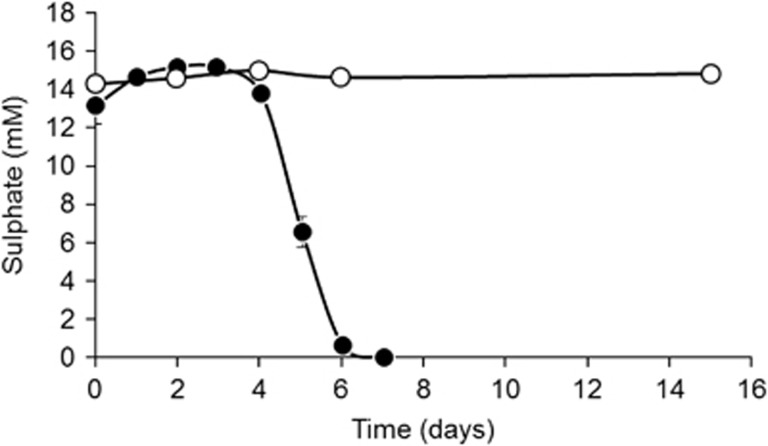
Tyne Estuary sediment slurry incubation following triple autoclaving at 121 °C for 20 min. Replicate slurries were incubated at 50 °C (closed symbols) and 80 °C (open symbols) and the sulphate concentration monitored over a period of 15 days. Slurries were amended with organic substrates (3 mM acetate, propionate, butyrate, lactate, glucose and 3 g l^−1^ tryptic soy broth). No sulphate removal occurred in slurries incubated at 80 °C. Error bars show s.e. (*n*=3).

Autoclave-surviving, sulphate-reducing bacteria were also present in sediments from the Tyne Estuary where rapid sulphate removal occurred in 50 °C sediment slurry incubations previously triple autoclaved (Figure 2). The more rapid sulphate reduction than in Portishead Estuary slurries (Figure 1) presumably was due to the organic substrates added to the Tyne Estuary slurries. However, similar to Portishead sediment incubations no sulphate reduction occurred in slurries incubated at 80 °C. The *in situ* temperature of the Tyne estuary fluctuates between 4 and 22 °C annually, so it is again unlikely that these bacteria are active *in situ*, and hence, they are probably present in these temperate sediments as dormant spores. These and the Tamar Estuary results show that the 1% Guaymas Basin sediment additional inoculum was probably not the source of the autoclave-surviving, sulphate-reducing bacteria.

### Physiology and identity of *Desulfotomaculum* sp. C1A60 from autoclaved Portishead Estuary sediments

**Figure 3:**
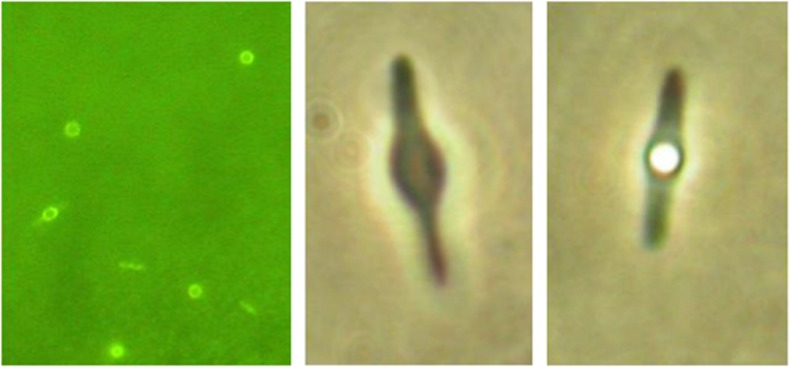
Photomicrographs of *Desulfotomaculum* sp. C1A60, including a cell with a central endospore.

Cultures of *Desulfotomaculum* sp. C1A60 contained rod-shaped cells, some of which formed central spherical endospores and appeared microscopically pure (Figure 3). Temperature range for growth, based on microscopic cell counts, for H_2_/CO_2_ as a substrate was 50–72 °C, for 2 mM fatty acid mixture plus H_2_/CO_2_ as a substrate was 58–69 °C and for 2 mM fatty acid mixture plus N_2_/CO_2_ was 54–69 °C. Similar results were obtained for activity based on sulphate removal or acetate production: 50–72 °C, 58–72 °C and 54–71 °C, respectively. Hence, different substrates have a limited effect on growth and activity temperature range. The minimum and optimum growth temperature of *Desulfotomaculum* sp. C1A60 is similar to that of *D. kuznetsovii*^T^ (minimum 50 °C, optimum 60–65 °C), however, the temperature maximum is considerably lower (Table 1). Although *Desulfotomaculum* sp. C1A60 sporulated well if grown with fatty acids, no spores were observed in cultures with lactate or hydrogen as electron donors. At temperatures above 60 °C, stationary phase cells quickly formed spheroblasts and disintegrated, irrespective of whether sporulation occurred.

**Figure 4:**
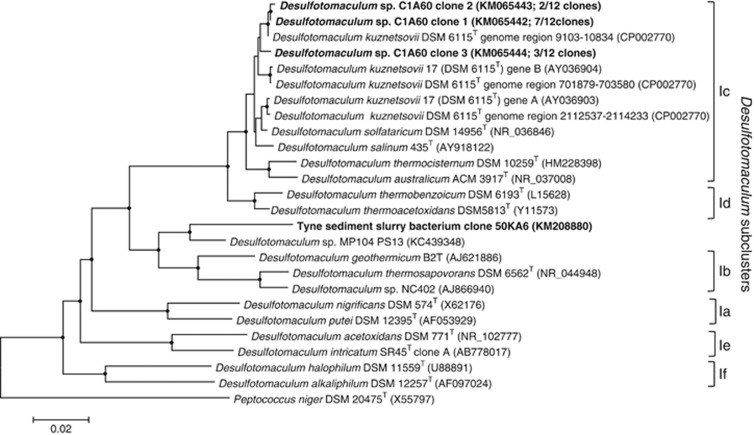
Phylogenetic tree showing the affiliation of the 16S rRNA gene sequences of heat-resistant *Desulfotomaculum* sp. C1A60 and River Tyne sediment slurry bacterium within the genus *Desulfotomaculum*. The tree was constructed with 1698 base positions of aligned 16S rRNA gene sequences and obtained using Minimum Evolution and LogDet distance with *Peptococcus niger* as an outgroup. Bootstrap support values over 50% (1000 replicates) are indicated by filled circles and bootstrap supported branches were present in all trees calculated using Minimum Evolution with LogDet, as well as neighbour-Joining with Jukes–Cantor and maximum likelihood with Tamura–Nei methods. Scale bar represents 2% sequence divergence. *Desulfotomaculum* subclusters are based on those described by Stackebrandt *et al.* (1997). *D. kuznetsovii* DSM 6115T and *D. kuznetsovii* 17 are the same type strain. All new sequences from this study are in bold, with their accession number and the number of clones for each different *Desulfotomaculum* sp. C1A60 16S rRNA gene given in parentheses.

Twelve individual 16S rRNA gene clone inserts from *Desulfotomaculum* sp. C1A60 were fully sequenced, and grouped into three distinct sequence types with variable length. Sequences also contain insertions characteristic of some *Desulfotomaculum* spp., that have been hypothesised to be involved in the operation of ribosomes at high temperature (Visser *et al.*, 2013), resulting in them being longer than typical 16S rRNA gene sequences: sequence 1 (1695 bp; 7 clone representatives), sequence 2 (1711 bp; 2 clone representatives) and sequence 3 (1676 bp; 3 clone representatives). No other sequences were detected. The three sequence types exhibited between 94% and 97% nucleotide sequence similarity to each other, and all exhibited closest sequence similarity to *D. kuznetsovii* DSM 6115^T^ (average ∼96%, accession number CP002770), grouping within the *Desulfotomaculum* subcluster Ic (Stackebrandt *et al.*, 1997, Figure 4). The presence of multiple divergent 16S rRNA genes seems to be a characteristic of bacteria related to *D. kuznetsovii* (Tourova *et al.*, 2001). For example, *D. kuznetsovii* DSM 6115^T^ (same strain as *D. kuznetsovii* strain 17) contains at least two divergent 16S rRNA genes (Figure 4). The complete genome sequence of *D. kuznetsovii* DSM 6115^T^ has shown that in fact it contains three copies of the 16S rRNA gene (Visser *et al.*, 2013): region 1 (1732 bp; 9103–10834 genome sequence location), region 2 (1702 bp; 701879–703580 genome sequence location) and region 3 (1697 bp; 2112537–2114233 genome sequence location). The *Desulfotomaculum* sp. C1A60 *dsrA* partial nucleotide sequence showed 99% sequence similarity to the *dsrA* gene within the genome of *D. kuznetsovii* DSM 6115^T^ (accession number CP002770) and 92–97% sequence similarity to *dsrA* genes of thermophilic sulphate-reducing bacteria from an underground heavy-metal mine (Nakagawa *et al.*, 2002). The next closest pure-culture match was with the *dsrA* gene of *D. thermocisternum* (AF074396) from hot, North Sea oil reservoir water (Nilsen *et al.*, 1996) with 89% sequence similarity.

### Triple-autoclave experiments with *Desulfotomaculum* spp

*Desulfotomaculum sp.* C1A60 grown on 2 mM fatty acid mix and containing both spores and vegetative cells survived triple autoclaving (Table 2). However, *Desulfotomaculum sp.* C1A60 grown on 20 mM lactate, had only visible vegetative cells and only survived two autoclavings (only limited growth after two autoclavings). It seems likely that survival of autoclaving by lactate-grown cultures was due to a small number spores present in this culture. *D. kuznetsovii*^T^ (Nazina *et al.*, 1989) and *D. geothermicum* B2T (Sass and Cypionka, 2004) both originally isolated from geothermal ground waters (Daumas *et al.*, 1988), also survived triple autoclaving. In contrast, *D. thermosapovorans*, *Desulfotomaculum* sp. NC402 and *D. acetoxidans*, did not survive autoclaving. However, they did survive pasteurization, suggesting that the original inoculating cultures did contain some spores. Repeating these autoclaving experiments in gas-tight glass crimp tubes (Bellco) instead of polypropylene Falcon tubes, to prevent any possibility of oxidation during treatment, produced the same results. This indicates that any oxidation of the media during autoclaving in Falcon tubes was not a factor in their subsequent lack of growth. Removal of sulphate and volatile fatty acids in triple-autoclaved cultures of *Desulfotomaculum* sp. C1A60 (limited acetate produced, ∼2.5 mM) and *D. kuznetsovii*^T^ confirmed activity as well as growth (data not shown). During autoclaving cultures reached a temperature maximum of 132 °C.

### High-temperature autoclaving and high-temperature survival experiments

Survival of *Desulfotomaculum* sp. C1A60 and *D. kuznetsovii*^T^ cultures was tested under increasingly high autoclave temperatures and times, up to 135 °C and 15 min (Table 3). *Desulfotomaculum* sp. C1A60 survived autoclaving at 130° for 15 min (both in Falcon and Bellco tubes, maximum temperature reached, 138 °C), but not autoclaving at 135 °C (max, 154 °C reached). In contrast, *D. kuznetsovii*^T^ cultures survived autoclaving at 135 °C for 15 min (max, 154 °C reached). As this was the upper safety limit of the autoclave, higher temperatures were tested in a heated oil bath, which also provided consistent and more accurate temperatures (Table 4). Consistent with the autoclave results *Desulfotomaculum* sp. C1A60 survived 15 min at 130 °C, but in addition, also survived at this temperature for 30, but not 60 min. *D. kuznetsovii*^T^ cultures survived exposure to 140 °C for 15 min, but not for 30 min or higher temperatures. Maximum temperatures of the oil bath, which allowed spore survival, were noticeably below the maximum temperatures experienced in the autoclave (Table 3), so length of exposure at maximum temperature may be more crucial than shorter periods at higher temperatures, for spore survival. Interestingly, survival temperatures in the autoclave and non-pressurised oil bath vials were not very different, suggesting that pressure on its own is not that important to limiting spore survival, assuming that pressure was higher during autoclaving.

### Viability of *Desulfotomaculum* sp. C1A60 spores and vegetative cells surviving pasteurisation or autoclaving

MPN counts of spore-only cultures of *Desulfotomaculum* sp. C1A60 were progressively reduced with successive autoclavings from 0.596 to 0.061 and 0.023% recovery (average of two MPN series) for one, two and three autoclavings, respectively (Table 5). In contrast, the MPN counts for the vegetative cell-only culture were markedly reduced by a single autoclaving, and after two and three autoclavings only a few cells survived. It is likely that a small number of spores or spore-containing cells in the vegetative cell culture, not detected microscopically, may have accounted for the limited growth after autoclaving. To account for 93 and 23 cells ml^−1^ surviving triple autoclaving by considering spore survival, would require 1.53 × 10^5^ and 1.02 × 10^5^ ml^−1^ spores, respectively, in the original vegetative cell culture. The ability of relatively few spores to grow in culture medium after autoclaving demonstrates the potential significance of even the survival of a small number of spores in the environment to exploit new growth opportunities (for example, using our spore survival data and MPN counts of spore-forming, thermophilic sulphate-reducing bacteria in Portishead sediments (average 2.9 × 10^4^ g^−1^ for top 25 cm of sediment, unpublished results) indicates that only 25 spores may have survived triple autoclaving of slurries, yet sulphate reduction began rapidly, Figure 1). This is reinforced by the much higher viability of spores, even without treatment compared with vegetative cells (20% compared with 0.7%, a factor of ∼30, Table 5).

### Summary and significance

The combined high survival and culturability of some *Desulfotomaculum* spores demonstrated in these experiments could explain why spore-forming bacteria often represent a considerable proportion of culturable diversity in marine sediments (Isaksen *et al.*, 1994), including in Portishead sediments (thermophilic, spore-forming, sulphate-reducing bacteria were 93% of the total pasteurised plus non-pasteurised MPN thermophilic, sulphate-reducing bacterial counts in the top 25 cm of sediment, unpublished results). The culturability of *Desulfotomaculum* spores is ∼30 times greater than that of their vegetative cells (Table 5, comparing direct spore/cell counts with MPN counts). If this reflects the general better culturability of spores compared with vegetative cells, then spore-forming bacteria may be over-represented in culture-based biodiversity surveys. This may be in contrast to their potential under-representation in culture-independent surveys reliant upon extraction of DNA from spores that may be more resistant to lysis than vegetative cells (Wunderlin *et al.*, 2014). The 50–72-°C growth range of *Desulfotomaculum* sp. C1A60 means that it cannot grow *in situ* in Portishead intertidal sediments, which have a maximum temperature of ∼23 °C. This situation is the same as observed for *Desulfotomaculum* sp. C1A60's closest relative *D. kuznetsovii* in other marine sediments (Isaksen *et al.*, 1994) and related types in Tyne Estuary sediments. Despite the thermophilic characteristics of these *Desulfotomaculum* species, they survive well, presumably as spores, in cold marine sediments, with an estimated half-life of ∼300 years (de Rezende *et al.*, 2013).

In addition, the high temperature and pressure (autoclave) tolerance of some *Desulfotomaculum* spores would enable them to survive and be dispersed via hot hydrothermal (Dhillon *et al.*, 2003; Wang *et al.*, 2009; Gerasimchuka *et al.*, 2010) crustal (Cowen *et al.*, 2003) and other fluid flow through deep hot sediments, with short exposures to temperatures up to even 140–150 °C (Tables 3 and 4 and an oil reservoir *Desulfotomaculum* sp. surviving 131 °C for 20 min, Rosnes *et al.*, 1991). High-temperature subsurface environments such as deep petroleum reservoirs could also be inoculated with spore-forming thermophilic *Desulfotomaculum* spp., (Aullo *et al.*, 2013) via natural fluid flows or for petroleum reservoirs during secondary oil recovery using seawater injection. Consistent with this situation, *D. kuznetsovii* (Nazina *et al.*, 1989) and *D. geothermicum* B2T (Sass and Cypionka, 2004), which both survived triple autoclaving, were originally isolated from geothermal ground waters (65 and 58 °C, respectively). Interestingly, *D. kuznetsovii* is the most robust in terms of high-temperature survival and *Desulfotomaculum* sp. C1A60 from Portishead sediments, which also survives triple autoclaving, is most closely related to this bacterium (∼96%). In contrast, the thermophilic, autoclave-surviving, sulphate reducer (50 °C) from Tyne estuary sediments (UK), is affiliated to a different *Desulfotomaculum* subgroup, which contains *D. geothermicum* (Figure 4). Clearly, for Portishead and probably other sediments, the sediment matrix does not contribute to the triple-autoclave survival of spores, as the *Desulfotomaculum* sp. C1A60 isolate alone also survives this and higher-temperature treatment (Tables 3 and 4).

Although some hyperthermophiles can survive autoclaving (for example, *Pyrolobus fumarii* temperature maximum 113 °C, survives 1-h autoclaving at 121 °C (Blochl *et al.*, 1997), it is surprising that a thermophile, with a much lower temperature maximum (∼72 °C), can survive the same and even higher-temperature autoclave conditions (up to 135 °C for 15 min autoclaving and 140 °C for 15 min in an oil bath—*D. kuznetsovii*, Tables 3 and 4). However, this extreme high-temperature survival is likely due to spores (Table 5), unlike the archaeon *Pyrolobus fumarii*, which does not form spores. Other spore-forming bacteria have occasionally been reported to survive autoclaving (Gong *et al.*, 2012), but not sequential double autoclaving (Thomas, 2006), whereas several *Desulfotomaculum* sp. do not even survive one autoclaving (Table 2). Therefore, the triple-autoclave-surviving *Desulfotomaculum* sp. C1A60, *D. kuznetsovii*, *D. geothermicum* and the Tyne sediment *Desulfotomaculum* sp. are rather unusual. Perhaps this is a feature that demonstrates that these thermophilic *Desulfotomaculum* do experience short exposure to high temperatures in their natural environments or during dispersal, which clearly suggests a high-temperature origin for these *Desulfotomaculum* spp. and possibly a subsurface origin considering the original source of both *D. kuznetsovii* and *D. geothermicum*. Although high-temperature-resistant *Desulfotomaculum* spp. appear to be common (for example, Portishead and Tyne Estuary sediments and presumably *D. kuznetsovii* from Aarhus Bay, Isaksen *et al.*, 1994), they are not universally distributed (for example, absent from Tamar Estuary sediments). A similar situation was found in a general survey of endospore-forming, thermophilic *Firmicutes* (Muller *et al.*, 2014) and differences in distributions were thought to be related to different connectivity of local water masses to ocean circulation.

The unique autoclave and temperature resilience of *D. kuznetsovii*-related *Desulfotomaculum* spp. probably reflects a general spore robustness that helps to explain the remarkable survival of some spores on geological timescales (for example, (Cano and Borucki, 1995; Vreeland *et al.*, 2000) and in deep, hot sediments (Parkes *et al.*, 2000, 2014). Research on *Bacillus* spp. has shown that a major factor determining resistance to wet-heat is the water content of the spore core, with lower core water content generally resulting in greater resistance (Setlow, 2006). During spore germination the core water content is elevated resulting in an increased sensitivity to heat (Setlow, 2014). Other factors contributing to heat resistance include the extent of spore core mineralisation, the sporulation temperature and spore DNA saturation with α/β-type small, acid-soluble proteins (Nicholson *et al.*, 2000).

The results presented here, may also have relevance to the conditions required for, and bacteria susceptible to, deep-burial sterilization, thought to be responsible for the occurrence of shallow non-biodegraded, hydrocarbon reservoirs, which are presently at relatively low temperatures (Wilhelms *et al.*, 2001); particularly, the ‘sterilizing' temperatures required may be higher for spore-forming bacteria, and some spore-forming bacteria may be particularly rapid re-colonisers. Also, survival after several serial autoclavings provides additional challenges when trying to identify biogenic versus thermogenic processes under high-temperature conditions, and highlights the need to consider longer and higher-temperature autoclaving for effective sterile controls (for example, Parkes *et al.*, 2011; Gong *et al.*, 2012, Tables 2 and 3) and sterility in general (Thomas, 2006).

## Figures and Tables

**Table 1:** *Desulfotomaculum* strains used in this study, their cultivation conditions, temperature range and origin

*Bacterial strain*	*Cultivation conditions*			*Temp. range*	*Origin*	*Reference*
	*Medium*	*Growth Temp.*	*Substrate*			
*Desulfotomaculum* sp. C1A60	Marine	58 °C	FA mix (2)	50–72 °C	Intertidal mudflat	This study
*D. kuznetsovii* DSM 6115^T^	Fresh	60 °C	FA mix (2)	50–85 °C	Geothermal water, 3000 mbsf	Nazina *et al.* (1989)
*D. geothermicum* B2T	Marine	45 °C	Lactate (20)	30–65 °C	Sandstone, 1060 mbsf	Sass and Cypionka (2004)
*D. thermosapovorans* DSM 6562^T^	Fresh	45 °C	Butyrate (10)	35–60 °C	Enrichment on rice hulls	Fardeau *et al.* (1995)
*Desulfotomaculum* sp. NC402	Fresh	45 °C	Lactate (20)	30–55 °C	Intertidal mudflat, 50 cmbsf	Sass *et al.* (2003)
*D. acetoxidans* DSM 771^T^	Fresh	25 °C	Acetate (20)	20–40 °C	Piggery waste	Widdel and Pfennig (1977)

Abbreviation: FA, fatty acid.

**Table 2:** Effect of pasteurisation and/or repeated autoclaving on survival of *Desulfotomaculum* sp. CIA60 and related *Desulfotomaculum* species (++=positive growth,+=limited growth, −−=no growth)

*Test strain*	*Autoclave conditions*	*Subculture*	*Pasteurisation*	*Times autoclaved*		
				*1*	*2*	*3*
*Desulfotomaculum* sp. C1A60 (spores and vegetative cells—grown on 2 mM fatty acid mix)	Falcon	++		++	++	++
*Desulfotomaculum* sp. C1A60 (microscopically vegetative cells only—grown on 20 mM sodium lactate)	Falcon	++		++	+	−−
*D. kuznetsovii*	Falcon	++		++	++	++
*D. geothermicum* B2T	Falcon	++	++	++	++	++
*D. thermosapovorans*	Falcon	++	++	−−	−−	−−
*D. thermosapovorans*	Bellco	++	++	−−	−−	−−
*Desulfotomaculum* sp. NC402	Falcon	++	++	−−	−−	−−
*Desulfotomaculum* sp. NC402	Bellco	+	++	−−	−−	−−
*D. acetoxidans*	Falcon	++	++	−−	−−	−−
*D. acetoxidans*	Bellco	++	++	−−	−−	−−

Falcon tubes are made from autoclavable polypropylene (not gas tight) and Bellco are glass tubes sealed with a septa (anaerobic).

**Table 3:** Effect of increasing autoclave temperatures and times on the survival of *Desulfotomaculum* sp. C1A60 and *D*. *kuznetsovii*

*Test strain*	*Autoclave programmed temperature*	*Time (min)*	*Autoclave tubes*	*Max. temperature (temperature indicator papers)*	*Growth—variable numbers of replicates positive subcultures*
*Desulfotomaculum* sp. C1A60	121 °C	15	Falcon	132 °C	++
	121 °C	30	Falcon	132 °C	++
	121 °C	60	Falcon	132 °C	++
	125 °C	15	Falcon	138 °C	++
	130 °C	15	Falcon	138 °C	−−− ++
	130 °C	15	Bellco	138 °C	++
	135 °C	15	Falcon	154 °C	−−−−−
	135 °C	15	Bellco	154 °C	−−
					
*D. kuznetsovii*	121 °C	15	Falcon	132 °C	++
	121 °C	30	Falcon	132 °C	++
	121 °C	60	Falcon	132 °C	++
	125 °C	15	Falcon	138 °C	++
	130 °C	15	Falcon	138 °C	++++++
	130 °C	15	Bellco	138 °C	++
	135 °C	15	Falcon	154 °C	+++
	135 °C	15	Bellco	154 °C	++

Number of subcultures that had positive growth (+) or no growth (−). Falcon tubes are made from autoclavable polypropylene (not gas tight) and Bellco are glass tubes sealed with a septa (anaerobic).

**Table 4:** Survival of *Desulfotomaculum* sp. C1A60 and *D. kuznetsovii* cultures at elevated temperature in an oil bath for varying time periods

*Test strain*	*Temperature*	*Time (min)*	*Growth—variable numbers of replicates positive subcultures (+)*
*Desulfotomaculum* sp. C1A60	121 °C	15	++
	125 °C	15	+++++
	130 °C	15	++++
	130 °C	30	+++
	130 °C	60	−−−
	135 °C	15	−−−−
	135 °C	30	−−−
	140 °C	15	−
	145 °C	15	−−
	150 °C	15	−−
*D. kuznetsovii*	121 °C	15	++
	125 °C	15	++
	130 °C	15	++
	135 °C	15	+++++
	140 °C	15	+++++
	140 °C	30	−−−
	140 °C	60	−−−
	145 °C	15	−−−−−
	145 °C	30	−−−
	145 °C	60	−−−
	150 °C	15	−−

**Table 5:** Culturability of *Desulfotomaculum* sp. C1A60 spores and vegetative cells, surviving pasteurisation or autoclaving based on replicate most probable number (MPN 1 and 2) estimates

*Treatment*	*Spore MPN 1 spore or cell count ml*^*−1*^	*Spore MPN 2 spore or cell count ml*^*−1*^
Original spore count	4.1 × 10^6^	4.1 × 10^6^
Untreated MPN count A	1.1 × 10^6^	2.9 × 10^4^
Untreated MPN count B	1.1 × 10^6^	1.1 × 10^6^
Pasteurized	1.1 × 10^6^	1.5 × 10^5^
Single autoclave	4.6 × 10^4^	2.9 × 10^3^
Double autoclave	4.6 × 10^3^	3.9 × 10^2^
Triple autoclave	1.1 × 10^3^	7.5 × 10^2^
Viable spores surviving triple autoclaving	0.027%	0.018%

	*Cell MPN 1 count ml*^*−1*^	*Cell MPN 2 count ml*^*−1*^
Original cell count	7.48 × 10^7^	7.48 × 10^7^
Untreated MPN count A	4.6 × 10^3^	3.9 × 10^3^
Untreated MPN count B	3.9 × 10^3^	2.1 × 10^3^
Pasteurized	1.1 × 10^3^	4.6 × 10^3^
Single autoclave	2.4 × 10^2^	1.1 × 10^3^
Double autoclave	93	75
Triple autoclave	23	93
Viable cells surviving triple autoclaving	0.000%	0.000%
